# *qRf8-1*, a Novel QTL for the Fertility Restoration of Maize CMS-C Identified by QTL-seq

**DOI:** 10.1534/g3.120.401192

**Published:** 2020-05-29

**Authors:** Mingmin Zheng, Tian Yang, Xiaowei Liu, Guihua Lü, Peng Zhang, Bin Jiang, Shufeng Zhou, Yanli Lu, Hai Lan, Suzhi Zhang, Chuan Li, Tingzhao Rong, Moju Cao

**Affiliations:** * Maize Research Institute, Sichuan Agricultural University, Chengdu 611130, China and; ^†^ Dongyang Maize Research Institute of Zhejiang Province, Zhejiang Academy of Agricultural Sciences, Dongyang 322100, China

**Keywords:** Maize, Cytoplasmic male sterility, Fertility restoration, Restorer gene, QTL-seq

## Abstract

C-type cytoplasmic male sterility (CMS-C), one of the three major CMS types in maize, has a promising application prospect in hybrid seed production. However, the complex genetic mechanism underlying the fertility restoration of CMS-C remains poorly understood. The maize inbred line A619 is one of the rare strong restorer lines carrying the restorer gene *Rf4*, but different fertility segregation ratios are found in several F_2_ populations derived from crosses between isocytoplasmic allonucleus CMS-C lines and A619. In the present study, the segregation ratios of fertile to sterile plants in the (CHuangzaosi × A619) F_2_ and BC_1_F_1_ populations (36.77:1 and 2.36:1, respectively) did not follow a typical monogenic model of inheritance, which suggested that some F_2_ and BC_1_F_1_ plants displayed restored fertility even without *Rf4*. To determine the hidden locus affecting fertility restoration, next-generation sequencing-based QTL-seq was performed with two specific extreme bulks consisting of 30 fertile and 30 sterile *rf4rf4* individuals from the F_2_ population. A major QTL related to fertility restoration, designated *qRf8-1*, was detected on the long arm of chromosome 8 in A619. Subsequently, *qRf8-1* was further validated and narrowed down to a 17.93-Mb genomic interval by insertion and deletion (InDel) and simple sequence repeat (SSR) marker-based traditional QTL mapping, explaining 12.59% (LOD = 25.06) of the phenotypic variation. Thus, using genetic analyses and molecular markers, we revealed another fertility restoration system acting in parallel with *Rf4* in A619 that could rescue the male sterility of CHuangzaosi. This study not only expands the original fertility restoration system but also provides valuable insights into the complex genetic mechanisms underlying the fertility restoration of CMS-C.

Cytoplasmic male sterility (CMS), a maternally inherited trait, is characterized by the inability to produce functional pollen and occurs widely in higher plants. Certain nuclear genes, termed restorers-of-fertility (*Rf*), can counteract the male sterile effect of the corresponding specific cytoplasm. CMS/*Rf* systems thus provide a useful genetic tool for the utilization of heterosis as well as a unique model for the elucidation of nuclear-cytoplasmic interactions in plants. To date, diverse *Rf* genes have been identified and characterized in various plants, and at least 15 *Rf* genes have been isolated from crop plants, which not only largely improves our knowledge of nuclear-cytoplasmic interactions but also facilitates the efficient utilization of CMS in practice (Bohra *et al.* 2016; Kim and Zhang 2018).

Maize is the first crop in which CMS was successfully used for the mass production of commercialized hybrid seeds (Kim and Zhang 2018). The various sources of CMS strains in maize can be divided into three major types: T (Texas), S (USDA), and C (Charrua) according to their differential fertility restoration patterns (Beckett 1971). CMS-T was once used extensively in maize hybrid seed production before the 1970s and was subsequently eliminated due to its susceptibility to the southern corn leaf blight epidemic caused by the fungus *Helminthosporium maydis* race T (Ullstrup 1972). In the case of CMS-T, two complementary dominant genes, *Rf1* and *Rf2*, separately located on chromosomes 3 and 9, are both required for complete fertility restoration (Wise and Schnable 1994; Laughnan and Gabay-Laughnan 1983). In addition, *Rf8* and Rf* can partially restore fertility to CMS-T in an *Rf2*/*Rf2* background (Dill *et al.* 1997). For CMS-S, a single dominant gene, *Rf3*, located on chromosome 2, is sufficient for fertility restoration (Laughnan and Gabay 1978; Kamps and Chase 1997; Tie *et al.* 2006; Zhang *et al.* 2006). In addition to this main restorer gene, other loci involved in the fertility restoration of CMS-S, such as *Rf9* (Gabay-Laughnan *et al.* 2009) and several quantitative trait loci (QTL) (Tie *et al.* 2006; Feng *et al.* 2015), have been reported. The male sterility of CMS-S is unstable and highly susceptible to environmental factors, which impedes its application in agriculture (Weider *et al.* 2009). By contrast, CMS-C with stable male sterility is currently widely used in maize hybrid seed production. Two dominant genes, *Rf4* and *Rf5*, which are located on chromosomes 8 and 5, respectively, can fully restore the fertility of CMS-C independently (Sisco 1991; Tang *et al.* 2001; Jaqueth *et al.* 2020). Furthermore, an inhibitor of *Rf5*, designated *Rf-I*, is mapped to chromosome 7, and it suppresses the function of *Rf5* but has no impact on *Rf4* in fertility restoration (Hu *et al.* 2006). *Rf4* was cloned and found to encode a basic helix-loop-helix (bHLH) transcription factor, and a single amino acid substitution controls fertility restoration (Jaqueth *et al.* 2020). However, the definite molecular mechanism of fertility restoration to CMS-C via *Rf4*, a transcription factor localized to the nucleus, remains elusive. In addition, *Rf6* (Qin *et al.* 1990), *Rfm_1_* (Chen and Chen 1992) and some QTL (Kohls *et al.* 2011) involved in the partial restoration of fertility to CMS-C have also been reported.

Although the fertility restoration system of CMS-C has been extensively studied since the 1970s, its underlying genetic mechanism still remains poorly understood. Moreover, many interesting phenomena found in previous studies suggest the complexity of fertility restoration to CMS-C. The maize inbred line A619 is widely used in studies of fertility restoration and was the first restorer line used to map the *Rf* gene for CMS-C. Some studies suggested the existence of only one restorer gene in A619, *Rf4*, located on the short arm of chromosome 8 (Kheyr-Pour *et al.* 1981; Tang *et al.* 2001). Sisco (1991) mapped *Rf4* to the same region and assumed that there might be another duplicate restorer gene on chromosome 3 in A619. Huang *et al.* (1997) mapped a dominant restorer gene of A619 to chromosome 7 and inferred that two restorer genes probably coexisted. In addition, a novel restorer gene, Rf*-A619, was discovered in A619 (Liu *et al.* 2016). The above research results imply that A619 probably rescues the male sterility of CMS-C via different mechanisms under different circumstances.

In the present study, we revealed another fertility restoration system of CMS-C in A619 acting in parallel with *Rf4* that could rescue the male sterility of CHuangzaosi. Genetic analyses demonstrated that multiple loci were involved in this fertility restoration system. Joint QTL-seq and traditional QTL mapping, a major QTL designated *qRf8-1*, was delimited to a 17.93-Mb genomic region on chromosome 8 and was associated with the fertility restoration of CMS-C under different environmental conditions. Our study not only further expands the original fertility restoration system but also provides valuable insights into the complex genetic mechanisms underlying the fertility restoration of CMS-C.

## Materials and Methods

### Plant materials and population construction

Five isocytoplasmic allonucleus CMS-C lines, namely, CHuangzaosi, C478, CMo17, C698-3 and C48-2, and five isonucleus allocytoplasmic CMS-C lines, namely, G48-2, EC48-2, ES48-2, RB48-2 and Lei48-2, were crossed with A619 to produce the respective F_1_ hybrids. The F_1_ hybrid obtained from the cross between CHuangzaosi and A619 was self-pollinated to generate F_2_ and F_2:3_ populations and consecutively backcrossed to Huangzaosi (normal cytoplasm) to generate BC_1_F_1_, BC_2_F_1_, BC_3_F_1_ and BC_6_F_1_ populations.

### Phenotyping male fertility

The degree of fertility was ranked on a scale of I to V using a modified Beckett’s scale (Beckett 1971) as follows: (I) no anthers exserted; (II) less than 25% anthers exserted, and no pollen or a small amount of pollen shed; (III) 25–50% anthers exserted, and some pollen shed; (IV) 51–75% anthers exserted, and some pollen shed; (V) more than 75% anthers exserted, and pollen shed normally. During the period of flowering, individual plants were carefully examined every two days, and a final fertility grade was assigned to each plant until the tassel dried. Thus, plants with grade I or II were recorded as sterile, while those with grades III to V were recorded as fertile.

In addition, the pollen fertility of the F_1_ hybrid was evaluated in three individual plants using the iodine-potassium iodide (I_2_-KI) staining method. Three non-dehiscent anthers were taken from the upper, middle and bottom portions of the tassel, and then the anthers were squashed and stained with 1% I_2_-KI solution on a glass slide. The pollen grains were subsequently observed under a light microscope (Zeiss Axio Imager M2), and three images of each replicate were taken for analysis.

Fertility ratings of the F_2_ and BC_1_F_1_ progenies were investigated in the spring of 2018 at Chengdu and in the fall of 2018 at Jinghong. Fertility ratings of the F_1_ hybrids and BC_2_F_1_ progenies were investigated in the fall of 2018 at Jinghong and in the spring of 2019 at Chengdu. Fertility ratings of the F_2:3_, BC_3_F_1_ and BC_6_F_1_ progenies were investigated in the spring of 2019 at Chengdu. All of the F_1_ hybrids and segregating populations were grown in a field, and field management was applied according to common agricultural practices.

### Development of an Rf4-targeted marker for genotyping

According to the genome sequence of GRMZM2G021276, primers F1/R1 (5′-CTGCATGAGCGTGTACCACT-3′, 5′-CCTTGTTTATATGTGGTCCGAA-3′) were designed to amplify *Rf4* in A619 and *rf4* in CHuangzaosi. Polymerase chain reaction (PCR) amplification was performed using KOD FX (TOYOBO) and the following recommended program: initial denaturation at 94° for 2 min, followed by 35 cycles of denaturation at 98° for 10 s, annealing at 58° for 30 s, extension at 68° for 2 min 30 s, and final extension at 68° for 5 min. Sequence alignment was conducted using DNAMAN software (Figure S1). Based on sequence variations in the third intron, the InDel marker B4-2 with primers F: 5′-CCTTGACCTCGTCCACCT-3′ and R: 5′-CATGGTAGCCTAGTACTCGT-3′ was designed to detect the genotype at the *Rf4* locus. PCR amplification was performed using 2× Taq Master Mix (TSINGKE) and the following program: initial denaturation at 95° for 5 min, followed by 35 cycles of denaturation at 95° for 30 s, annealing at 57° for 30 s, extension at 72° for 40 s, and final extension at 72° for 5 min. Amplified fragments were visualized in a 3.5% agarose gel with 0.5× TBE buffer and GoldView staining.

### Illumina sequencing and QTL-seq analysis

Based on the results of fertility rating and genotyping of the F_2_ population, two contrasting bulks, a fertility restored bulk (FR-bulk) and a sterility maintained bulk (SM-bulk), were constructed by mixing equal amounts of leaf tissues of 30 fully fertile *rf4rf4* plants (grade V) and 30 sterile *rf4rf4* plants (grade I or II) from the 2018 fall experiment. Total genomic DNA was isolated from two bulks as well as the parents, CHuangzaosi and A619, using a modified cetyltrimethylammonium bromide (CTAB) method (Porebski *et al.* 1997). Sequencing libraries were prepared using TruSeq Sample Preparation Kits following the manufacturer’s instructions, and then the qualified libraries were sequenced on the Illumina HiSeq X Ten platform to produce paired-end 150 bp (PE150) reads with a sequencing depth of 10× for the parental lines and 30× for each bulk.

After removing adapters and low-quality reads (reads containing unknown nucleotides > 10%, reads containing more than 50% bases with a *Q* value ≤ 5), the quality of the clean reads was further checked using the FastQC program (v. 0.19.7). The high-quality clean reads were subsequently aligned to B73_RefGen_v4 (www.maizegdb.org) using Burrows-Wheeler Aligner (BWA) software, and duplicates were marked using SAMtools (Li and Durbin 2009). Genome Analysis Toolkit (GATK) v3.3 was used to call single nucleotide polymorphisms (SNPs) and InDels across parental lines and bulks (McKenna *et al.* 2010).

Homozygous SNPs between parental lines with high quality (sequence read depth > 5×) were selected for SNP-index analysis. The SNP-index, which represented the proportion of reads harboring the A619 genotype at every SNP site, was calculated for the FR and SM bulks (Abe *et al.* 2012; Takagi *et al.* 2013). Thus, the SNP-index was defined as 0 if the entire short sequence reads were derived from CHuangzaosi and 1 if the entire short sequence reads were derived from A619. In practice, the loci with read depth < 7 or with SNP-index < 0.3 or > 0.7 in both bulks were filtered out to eliminate false positives. Next, the Δ(SNP-index) was calculated by subtracting the SNP-index of the SM-bulk from that of the FR-bulk (Takagi *et al.* 2013). The average SNP-index of SNPs physically mapped across ten chromosomes was calculated using a sliding window approach with a 5-Mb window size and 10-kb increment. The SNP-index graphs for each bulk and the corresponding Δ(SNP-index) graph were plotted. Using a null hypothesis of no QTL, we calculated the statistical confidence intervals of the ∆(SNP-index) with given read depths (Takagi *et al.* 2013; Klein *et al.* 2018). Regions in which the average Δ(SNP-index) of a locus was significantly larger (*P* < 0.05) than the threshold were selected as candidate intervals associated with fertility restoration.

### Validation and mapping of the candidate region

To verify the QTL associated with fertility restoration identified by QTL-seq, traditional QTL analysis was performed using fertility rating data obtained from the F_2_ population. SSR markers in and around the predicted QTL region of chromosome 8 (www.maizegdb.org) were employed for polymorphism screening between the two parental lines. Additional InDel markers in the same region were developed using the whole-genome resequencing data of the two parental lines. Polymorphic markers with strong specificity were applied to genotype the 949 *rf4rf4* individuals of the F_2_ population. Based on the genotype data of the F_2_ population, a genetic linkage map was constructed using QTL IciMapping V4.1 (http://www.isbreeding.net/). QTL analysis was conducted using the Inclusive Composite Interval Mapping of Additive (ICIM-ADD) module within QTL IciMapping V4.1 (Meng *et al.* 2015). The scanning step was set at 1.0 cM, and the probability in the stepwise regression was set at 0.001. The logarithm of odds (LOD) threshold to accept the presence of a QTL was calculated using 1,000 permutation tests (*P* < 0.05).

### Data availability

The authors affirm that all data necessary for confirming the conclusions of the article are present within the article, figures and tables. Supplemental material available at figshare: https://doi.org/10.25387/g3.12277268.

## Results

### Evaluation of the fertility restoration capability of A619

**Figure 1:**
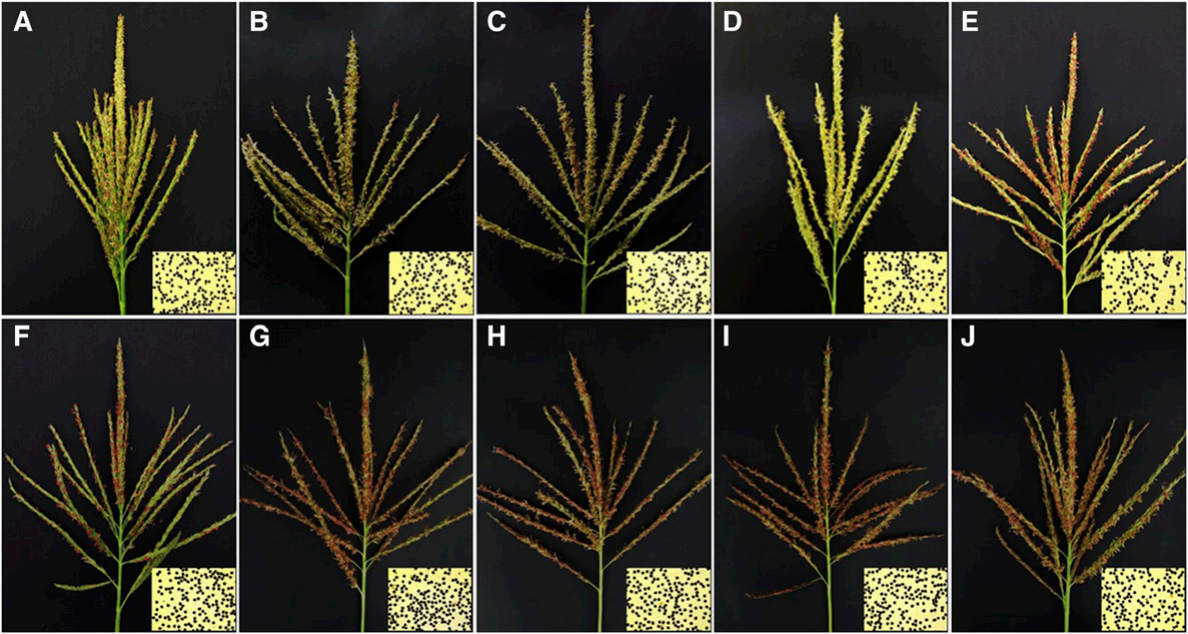
Fertility performance of F_1_ hybrids crossed between CMS-C lines and A619. (A) CHuangzaosi × A619, (B) C478 × A619, (C) CMo17 × A619, (D) C698-3 × A619, (E) C48-2 × A619, (F) G48-2 × A619, (G) EC48-2 × A619, (H) ES48-2 × A619, (I) RB48-2 × A619, (J) Lei48-2 × A619.

Individuals of all F_1_ hybrids obtained from the crosses between ten CMS-C lines and A619 were fully male-fertile with an identical fertility grade of V. The pollen stainability of the F_1_ hybrids ranged from 93.68 to 98.82% in different environments (Figure 1 and Table S1). A619 completely restored the fertility of all CMS-C lines tested in this study. Combined with previous research (Kheyr-Pour *et al.* 1981; Sisco 1991; Tang *et al.* 2001; Liu *et al.* 2016), we can conclude that A619 is capable of fully restoring the fertility of all CMS-C lines tested so far, regardless of the subtypes of cytoplasm and nuclear backgrounds, indicating the strong and broad-spectrum fertility restoration capability of A619 to CMS-C lines.

### Genetic analysis of fertility restoration in the (CHuangzaosi × A619) F_2_ and BC_1_F_1_ populations

A619 is known as a CMS-C restorer line with a dominant restorer gene (*Rf4*) mapped on the short arm of chromosome 8 (Sisco 1991; Tang *et al.* 2001), but this does not preclude the existence of other restorer genes. To further explore the restoration mechanism of A619, the fertility of all individuals in the F_2_ and BC_1_F_1_ populations derived from the cross between CHuangzaosi and A619 was investigated in the spring of 2018 at Chengdu and in the fall of 2018 at Jinghong (Table 1). Aberrant segregation ratios of fertile to sterile plants were observed in the F_2_ (Chengdu: 36.76:1, Jinghong: 36.78:1) and BC_1_F_1_ (Chengdu: 2.34:1 and Jinghong: 2.40:1) populations. The fertility segregation ratios did not follow a common monogenic or digenic pattern of inheritance of fertility restoration to CMS-C. *Rf4* could not explain every scenario of fertility restoration to CMS-C in this study. Interestingly, only 102 of the 3,853 plants in the F_2_ population were sterile, whereas the sterile plants accounted for 29.78% of the total plants in the BC_1_F_1_ population.

**Table 1:** Fertility ratings of F_2_ and BC_1_F_1_ progenies derived from the cross between CHuangzaosi and A619

Environments	Populations	Total plants	Plants of each fertility grade					Fertile plants	Sterile plants	Observed ratio
			I	II	III	IV	V			
2018, spring, Chengdu	F_2_	1246	5	28	29	10	1174	1213	33	36.76:1
	BC_1_F_1_	474	45	97	34	14	284	332	142	2.34:1
2018, fall, Jinghong	F_2_	2607	11	58	38	25	2475	2538	69	36.78:1
	BC_1_F_1_	238	24	46	7	3	158	168	70	2.40:1

Based on three deletions (15 bp, 20 bp and 1 bp) found in the third intron of *rf4* in CHuangzaosi compared to *Rf4* in A619, the InDel marker B4-2 was developed to detect the genotype at the *Rf4* locus. As expected, a 316-bp fragment of *Rf4* was amplified in A619, while a 280-bp fragment of *rf4* was amplified in CHuangzaosi. Subsequently, a total of 3,853 F_2_ plants and 712 BC_1_F_1_ plants were genotyped by the InDel marker B4-2 (Table 2). The InDel marker B4-2 showed a good fit to the expected 1:2:1 segregation ratio in the F_2_ populations (Chengdu: *χ^2^* = 2.16, *P* = 0.34; Jinghong: *χ^2^* = 0.98, *P* = 0.61) and 1:1 segregation ratio in the BC_1_F_1_ populations (Chengdu: *χ^2^* = 2.44, *P* = 0.12; Jinghong: *χ^2^* = 2.03, *P* = 0.15) for a single gene model. A total of 2,901 *Rf4*-containing (*Rf4Rf4* or *Rf4rf4*) plants were detected in the F_2_ population, of which 2,891 plants were fully fertile (grade V) and the other ten plants were partially fertile (grade III or IV). While 952 *rf4rf4* plants in the F_2_ population displayed varying degrees of fertility, of which 758 plants were fully fertile (grade V), 92 plants were partially fertile (grade III or IV) and 102 plants were sterile (grade I or II). For the BC_1_F_1_ population, there were 384 *Rf4rf4* plants, of which 383 plants were fully fertile (grade V) and only one plant was partially fertile (grade IV). While 328 *rf4rf4* plants displayed varying degrees of fertility, of which 59 plants were fully fertile (grade V), 57 plants were partially fertile (grade III or IV) and 212 plants were sterile (grade I or II). Completely and partially restored *rf4rf4* plants were frequently observed in the F_2_ and BC_1_F_1_ populations. Notably, approximately 79.62% of the *rf4rf4* plants in the F_2_ population were fully fertile, whereas the percentage of fully fertile *rf4rf4* plants in the BC_1_F_1_ population was considerably reduced (17.99%). Thus, the above results imply that another fertility restoration system acting in parallel with *Rf4* in A619 is capable of fully or partially restoring the fertility of CHuangzaosi independently.

**Table 2:** The segregation of fertility and B4-2 marker genotypes in F_2_ and BC_1_F_1_ populations derived from the cross between CHuangzaosi and A619

Populations	Genotypes	Plants of each fertility grade					Fertile plants	Sterile plants	Total plants	Theoretical ratios	*χ^2^*	*P* value	Environments
		I	II	III	IV	V							
F_2_	*Rf4Rf4*	0	0	4	1	322	327	0	327	1:2:1	2.16	0.34	2018, sping, Chengdu
	*Rf4rf4*	0	0	3	0	625	628	0	628				
	*rf4rf4*	5	28	22	9	227	258	33	291				
	*Rf4Rf4*	0	0	0	0	630	630	0	630	1:2:1	0.98	0.61	2018, fall, Jinghong
	*Rf4rf4*	0	0	2	0	1314	1316	0	1316				
	*rf4rf4*	11	58	36	25	531	592	69	661				
BC_1_F_1_	*Rf4rf4*	0	0	0	1	253	254	0	254	1:1	2.44	0.12	2018, sping, Chengdu
	*rf4rf4*	45	97	34	13	31	78	142	220				
	*Rf4rf4*	0	0	0	0	130	130	0	130	1:1	2.03	0.15	2018, fall, Jinghong
	*rf4rf4*	24	46	7	3	28	38	70	108				

### Genetic inheritance pattern of the novel fertility restoration system

To further characterize the inheritance pattern of the novel fertility restoration system, a set of progenies derived from the (CHuangzaosi × A619) F_2_ and BC_1_F_1_ populations were systemically analyzed. *Rf4rf4* plants with fertility grade V and *rf4rf4* plants with fertility grades II to IV in the BC_1_F_1_ population were randomly selected to backcross with Huangzaosi, and the fertility of the BC_2_F_1_ progenies was evaluated during 2018-2019 (Table S2). For BC_2_F_1_ progenies derived from fully restored *Rf4rf4* plants, one ear gave a good fit to a 1 fertile:1 sterile ratio (*χ^2^* = 1.05, *P* = 0.31). However, the other ear deviated from the 1:1 ratio (*χ^2^* = 12.83, *P* = 0.00), probably due to the effect of the other fertility restoration system. By contrast, 490 of 561 BC_2_F_1_ individuals (approximately 87.34%) derived from fully restored *rf4rf4* plants were sterile. Only five BC_2_F_1_ individuals derived from fully restored *rf4rf4* plants were rated grade V, and 66 BC_2_F_1_ individuals derived from fully restored *rf4rf4* plants were rated grade III or IV. Approximately 35.37% of *rf4rf4* individuals in the BC_1_F_1_ population were fully or partially fertile, but the proportion of fertile individuals in the BC_2_F_1_ population derived from fully restored *rf4rf4* plants was considerably reduced (approximately 12.66%). BC_2_F_1_ progenies derived from partially restored *rf4rf4* plants were partially fertile or sterile, and partially fertile individuals accounted for only 3.65% of the total individuals in this population. All BC_2_F_1_ progenies derived from sterile *rf4rf4* plants maintained male sterility. Additionally, BC_3_F_1_ and BC_6_F_1_ progenies derived from *Rf4rf4* plants gave a satisfactory fit to a 1 fertile:1 sterile ratio. Furthermore, we found many fully or partially restored F_2:3_ progenies generated from *rf4rf4* plants (Table S2). All these results collectively suggest that a number of QTL are probably involved in this complex genetic system.

### QTL-seq identified a major QTL controlling fertility restoration

**Figure 2:**
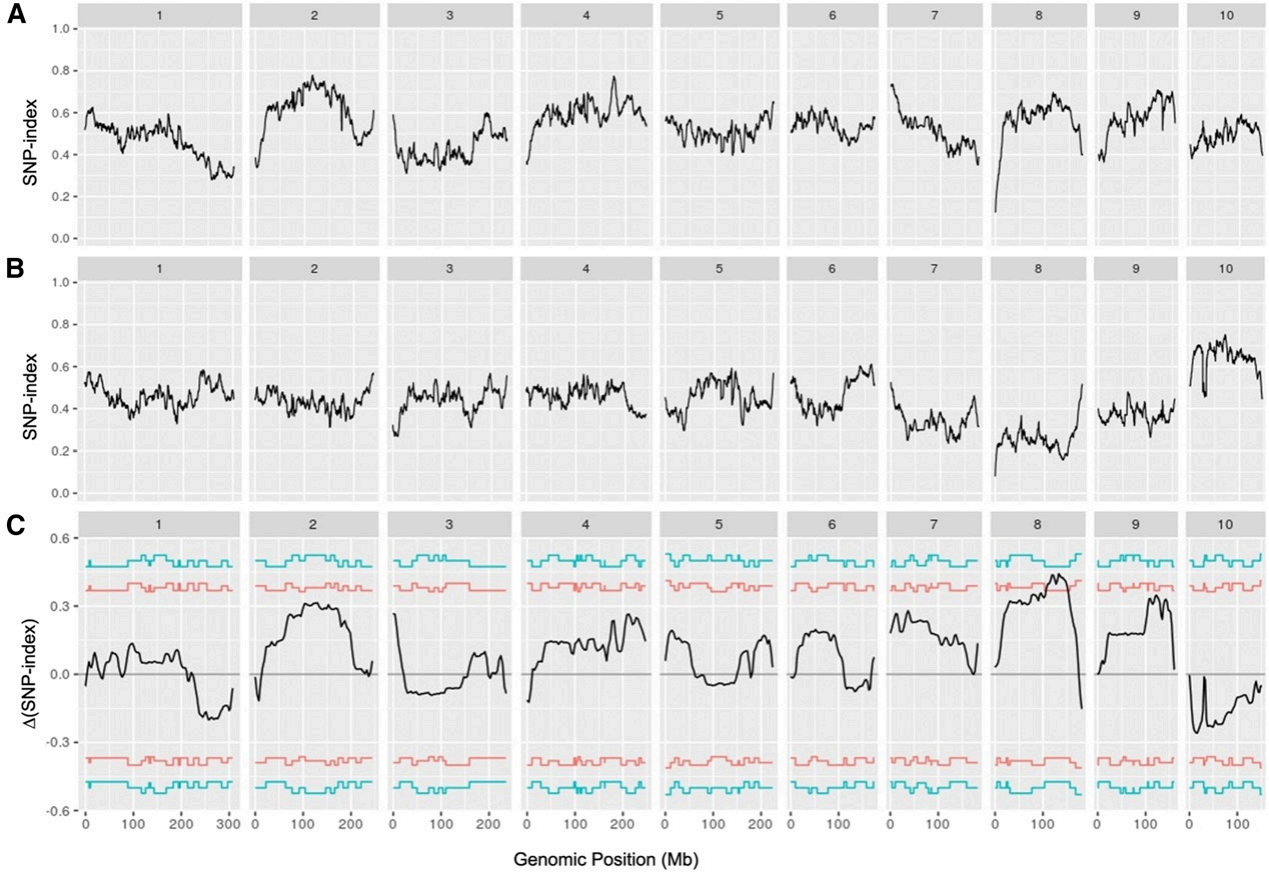
SNP-index graphs of FR-bulk (A), SM-bulk (B) and ∆(SNP-index) graph (C) from QTL-seq analysis of the F_2_ population. The *X*-axis represents the physical position (Mb) of ten maize chromosomes, and the *Y*-axis represents the SNP-index, which was calculated based on 5-Mb interval with a 10-kb sliding window. The ∆(SNP-index) graph (C) was plotted with statistical confidence interval under the null hypothesis of no QTL (red line for *P* < 0.05, green line for *P* < 0.01). A candidate QTL q*Rf8-1* was identified on chromosome 8 (117.01-142.85 Mb interval) using the criteria of SNP-index near 1 and 0 in FR-bulk (A) and SM-bulk (B), respectively, and the Δ(SNP-index) (C) above the confidence value (*P* < 0.05).

High-throughput sequencing using the Illumina platform resulted in 164,952,536, 149,935,224, 489,116,838 and 534,069,350 clean reads (150 bp in length) from CHuangzaosi (average depth 10.53×), A619 (average depth 9.71×), FR-bulk (average depth 31.22×) and SM-bulk (average depth 34.09×), respectively, covering 84.31–93.10% of the B73 reference genome (Table 3). The comparative genome sequence analysis of the two parents and two bulks ultimately identified 5,646,729 high-confidence SNPs. To identify the other candidate genomic regions responsible for fertility restoration in addition to *Rf4*, the SNP-index of each SNP locus was calculated using high-confidence SNPs. The average SNP-index across a 5-Mb genomic interval was computed individually in FR-bulk and SM-bulk using a 10-kb increment, and SNP-index graphs were generated by plotting the average SNP-index against all ten chromosomes of the B73 reference genome (Figure 2). Similarly, the Δ(SNP-index) was then calculated by integrating the SNP-index information of the two extreme bulks and plotted against the genomic positions (Figure 2). A genomic region on chromosome 8 ranging from 117.01 to 142.85 Mb exhibited unequal contributions from parents in both bulks. The average SNP-index of FR-bulk in this region was higher than 0.61 (the highest was 0.69), while the average SNP-index in the corresponding region of SM-bulk was lower than 0.25 (the lowest was 0.16). Moreover, at the 95% confidence level, the ∆(SNP-index) value of this genomic region was significantly different from 0. These results demonstrate that there is a major QTL controlling fertility restoration in the 117.01-142.85 Mb region on chromosome 8. This QTL was designated as *qRf8-1* according to the standard nomenclature suggested by Wang *et al.* (2017), which differed from the weak restorer factor *Rf8* for CMS-T located on the long arm of chromosome 2 (Meyer 2010).

**Table 3:** Summary of the sequencing data

Sample	Clean base (Gb)	GC content (%)	Q20 (%)	Q30 (%)	Average depth of sequencing	Genome coverage rate (%)	Mapping rate (%)
CHuangzaosi	24.68	46.00	97.41	92.48	10.53×	84.38	97.92
A619	22.42	47.25	97.44	92.59	9.71×	84.31	97.92
FR-bulk	73.17	47.85	97.09	91.90	31.22×	93.10	97.70
SM-bulk	79.89	46.02	97.44	92.54	34.09×	93.01	96.59

### Validation of the candidate region by traditional QTL mapping

**Figure 3:**
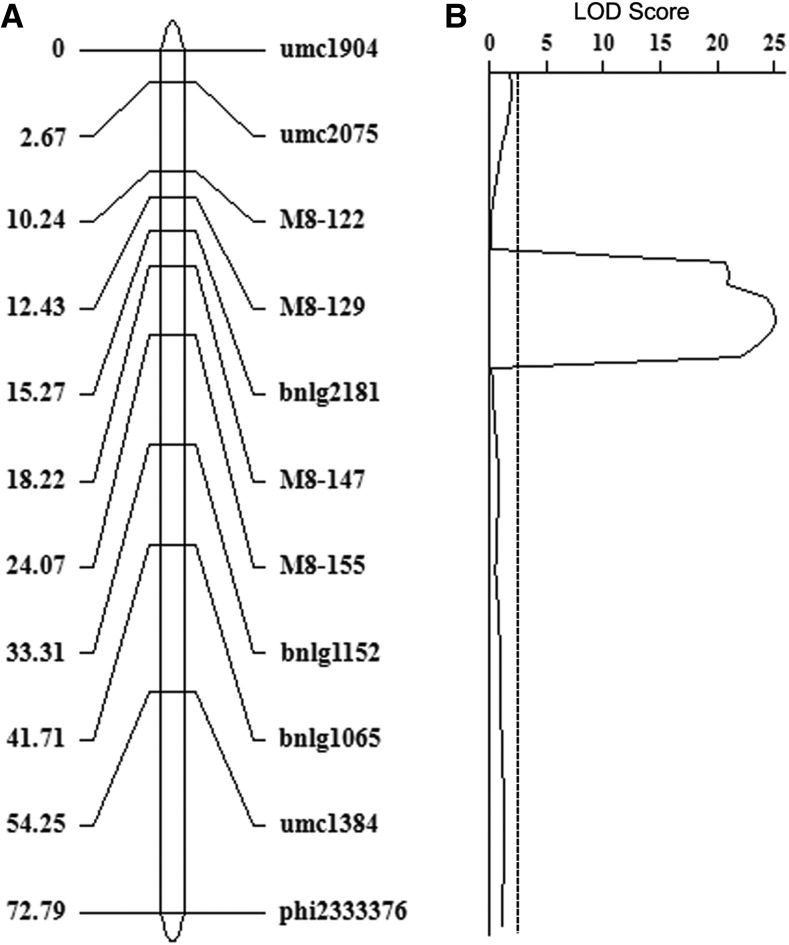
Validation of the fertility restoration QTL *qRf8-1* on chromosomes 8 using the F_2_ population. (A) Genetic map of 11 markers on maize chromosome 8. (B) Traditional QTL analysis confirmed the location of *qRf8-1* with flanking markers bnlg2181 and M8-155. The significance threshold (*P* < 0.05) is indicated by the horizontal dotted line.

To check the accuracy of the QTL for fertility restoration detected by QTL-seq, we performed traditional biparental QTL mapping. A total of 11 markers, including five SSR and six InDel markers (details in Table S3), were applied to genotype 949 *rf4rf4* plants selected from the F_2_ population in the 2018 spring and fall experiments. Based on the inclusive composite interval mapping (ICIM) method, a major QTL was mapped in the interval of bnlg2181 to M8-155 (Figure 3), which was physically located in the region of 137.74-155.67 Mb on the long arm of chromosome 8. This region with a maximum LOD score of 25.06 could explain 12.59% of the total phenotypic variance. The conventional QTL mapping result was consistent with that of the QTL-seq analysis and further narrowed down the *qRf8-1* locus to a 17.93-Mb genomic interval between bnlg2181 and M8-155. Moreover, we used the InDel marker M8-147 with the highest LOD score in this region to genotype 328 *rf4rf4* plants in the BC_1_F_1_ population. The InDel marker M8-147 showed a good fit to the expected 1:1 segregation ratio (*χ^2^* = 0.00, *P* = 1.00). We found that 82 of 116 completely or partially restored plants possessed A619 alleles in this population. These results further confirm the effects of *qRf8-1* on fertility restoration and could be useful in the marker-assisted selection of new male sterile and restorer lines with the C-type cytoplasm.

## Discussion

### Complex fertility restoration system of CMS-C in maize

CMS-C is one of the most attractive resources for maize hybrid production. Due to a limited understanding of the fertility restoration system and insufficient number of main restorer genes identified, the application of maize CMS-C is severely hampered. To date, two acknowledged dominant restorer genes, *Rf4* and *Rf5*, can fully restore the fertility of CMS-C (Kheyr-Pour *et al.* 1981; Tang *et al.* 2001; Jaqueth *et al.* 2020). The inhibitor of *Rf5* limits its application in practice (Hu *et al.* 2006), and *Rf4* is still the preferential choice to develop restorer lines by backcrossing. In the present study, we discovered an additional fertility restoration system acting in parallel with *Rf4* in A619 that could rescue the male sterility of CHuangzaosi independently. These findings confirm the earlier assumption that in addition to *Rf4* and *Rf5*, there is another parallel and independent genetic system with the same phenotypic effect on the fertility restoration of CMS-C (Vidakovic *et al.* 1997).

### The genetic background of CMS-C lines plays an important role in fertility restoration

A619 has the capability of fully restoring the fertility of all CMS-C lines tested so far and is considered a good donor for cultivating new restorer lines. Interestingly, when isocytoplasmic allonucleus CMS-C lines were used as female parents to study the fertility restoration of A619, conflicting results were reported. Some studies suggested that there is only one single restorer gene (*Rf4*) in A619 (Kheyr-Pour *et al.* 1981; Tang *et al.* 2001), but other studies inferred that other restorer genes probably coexist (Huang *et al.* 1997; Liu *et al.* 2016). In this study, we found that in addition to *Rf4* in A619, many QTL were also involved in the fertility restoration of CMS-C. It is interesting that similar events also occurred in other CMS-C restorer lines. For example, Fengke1 exhibited one restorer gene when crossed with CHuangzaosi and Cernan24, but it appears to have two restorer genes for CB37, CMo17 and C237 (Chen *et al.* 1979; Chen and Duan 1986; Tang *et al.* 2001). Therefore, the number and location of restorer genes in a given restorer line are applicable with respect only to the specific CMS-C line used as the tester. Different features of pollen abortion were observed in isocytoplasmic allonuclear CMS-C lines (Chen and Duan 1988), which suggested that male sterility might be restored through different mechanisms in specific nuclear genetic backgrounds. Moreover, the presence or absence of different minor restorer genes or modifying genes in CMS-C lines also influenced fertility restoration performance. More interestingly, similar phenomena were also reported in other crop plants, such as wheat (Xie *et al.* 1999) and rice (Zhou *et al.* 1986). Taken together, these results indicate that the genetic backgrounds of CMS lines commonly influence the function of restorer genes (Liu *et al.* 2016).

### Multiple genes involved in the novel fertility restoration system

The novel fertility restoration system is much more complex than the previously well-known fertility restoration system controlled by dominant main restorer genes. In this system, multiple loci might be involved in the fertility restoration of CMS-C, and only a certain combination of QTL could lead to the full or partial restoration of fertility. In this study, we employed QTL-seq (Takagi *et al.* 2013) to identify QTL associated with fertility restoration in a specific F_2_ population. Unfortunately, QTL-seq analysis only identified the QTL region *qRf8-1* on the long arm of chromosome 8, which was verified by traditional QTL mapping. A minor-effect QTL in bin8.06 associated with the partial restoration of CMS-C was reported in a previous study (Kohls *et al.* 2011). The position of *qRf8-1* was further narrowed down to a 17.93-Mb genomic interval between bnlg2181 and M8-155, but this locus explained only 12.59% of the phenotypic variance. Many F_2:3_ progenies generated from fully restored *rf4rf4* plants were fully or partially fertile. The fertility restoration related QTL alleles contributed from A619 were gradually lost during backcrossing, and therefore, only a small proportion of BC_2_F_1_ progenies generated from fully restored *rf4rf4* plants were fully or partially fertile. Some other QTL for fertility restoration of CMS-C were not detected in the present study, probably due to insufficient individuals in the two contrasting bulks or the detection limit of QTL-seq. According to the ∆(SNP-index) graph, minor-effect QTL might exist on chromosomes 2 and 9, which needs to be confirmed in further studies. It is worth noting that the fertility restoration of CMS-C displays many features of quantitative traits, but it is not exactly the same. Moreover, the fertility restoration of CMS is controlled not only by nuclear genes but also by cytoplasm genes. These features might influence the efficiency of QTL detection. In the future, it is imperative to identify and locate more restorer genes, which would be of great use in the development of high-quality male sterile, maintainer and restorer lines of maize. In summary, these results not only expand the original fertility restoration system but also contribute to a better understanding of the complexity of the fertility restoration mechanism for CMS-C.
